# miR-181a-regulated pathways in T-cell differentiation and aging

**DOI:** 10.1186/s12979-021-00240-1

**Published:** 2021-06-15

**Authors:** Chulwoo Kim, Zhongde Ye, Cornelia M. Weyand, Jörg J. Goronzy

**Affiliations:** 1 Department of Microbiology, Institute for Viral Diseases, Korea University College of Medicine, Seoul, Republic of Korea; 2 Division of Immunology and Rheumatology, Department of Medicine, Stanford University, Stanford, CA USA; 3 Department of Medicine, Palo Alto Veterans Administration Healthcare System, Palo Alto, CA USA

**Keywords:** microRNA, miR-181a, T cell aging, T cell differentiation, T cell activation, Memory T cells, Vaccine, Infectious disease, Replication stress

## Abstract

MicroRNAs (miRNAs) are regulatory noncoding RNAs important for many aspects of cellular processes including cell differentiation and proliferation. Functions of numerous miRNAs have been identified in T cells, with miR-181a regulating T cell activation thresholds during thymic T cell development and during activation of peripheral T cells. Intriguingly, miR-181a is implicated in defective antiviral and vaccine responses in older individuals, as its expression declines in naïve T cells with increasing age. Here, we review the pathways that are regulated by miR-181a and that explain the unique role of miR-181a in T cell development, T cell activation and antiviral T cell responses. These studies provide a framework for understanding how a decline in miR-181a expression in T cells could contribute to age-related defects in adaptive immunity. We furthermore review the mechanisms that cause the age-related decline in miR-181a expression and discuss the potential of restoring miR-181a expression or targeting miR-181a-regulated pathways to improve impaired T cell responses in older individuals.

## Background

microRNAs (miRNAs) are small noncoding RNAs that regulate gene expression post-transcriptionally through translational repression or mRNA degradation. By targeting many genes involved in common regulatory pathways, they function as crucial modulators of fundamental biological processes including cell development and differentiation [1]. Early studies with T cell-specific miRNA-deficient mice found alterations in T cell differentiation, cytokine production, proliferation and survival [2, 3]. Importance and function of individual miRNA in T cell responses have also been identified [4]. For example, miR-146a negatively regulates nuclear factor-κB (NF-κB) signaling induced by T cell receptor (TCR) activation [5]. The miR-17 ~ 92 cluster is important for the differentiation of effector CD8 T cells as well as T follicular helper cells [6, 7]. miR-155 is required for optimal CD8 T cell responses to viral infections and cancer [8].

The ability to mount protective immune responses against infections declines with age, resulting in increased mortality and morbidity from infections and cancer as exemplified by the influenza virus and SARS-CoV-2 infections [9, 10]. While vaccinations contribute to prevent infectious diseases in children and young adults, they are only moderately effective in older individuals [11]. The increased susceptibility to infections as well as the poor vaccine efficacy in the aged population are indicative of defective adaptive immunity including T cell and B cell responses [12, 13]. Given the important role of miRNAs in T cell immunity [4, 14], age-associated changes in miRNA networks could account for some of these functional deficits seen in older individuals. Indeed, expression of multiple miRNAs changes with increasing age, such as upregulation of miR-146a and miR-155 in the naive CD8 T cell compartment [15] and downregulation of multiple miRNAs in terminally-differentiated effector CD8 T cells [16]. Also, miR-21 that is induced after T cell activation, is expressed at higher amount in naïve CD4 T cells from older than young adults [17]. Aberrant miR-21 overexpression leads to sustained activation of several signaling pathways downstream of the TCR by repressing negative regulators, which in turn biases T cell differentiation toward inflammatory effector cells over T follicular helper cells and memory precursor cells in older individuals [17–19].

The miR-181 family is evolutionally conserved across all vertebrates. It comprises four nearly identical mature miRNAs (miR-181a, miR-181b, miR-181c, and miR-181d) from three clusters on separate chromosomes; miR-181ab1, miR-181ab2 and miR-181cd. The mature members of the miR-181 family have the identical 5′ seed sequence that determines binding to 3′ untranslated region (UTR) of their mRNA targets, suggesting their functional redundancy in targeting a similar set of genes. The miR-181 family is one of the most abundant miRNAs in lymphoid tissue [20]. Importance of miR-181a expression has been described during B cell development in the bone marrow [21, 22] and regulation of innate immune cell function such as macrophages and dendritic cells [23, 24]. Nevertheless, functions of miR-181a are best studied in T cells. miR-181ab1 deletion completely abrogated mature miR-181a expression in the thymus, while miR-181ab2 or miR-181cd deletion had no effects, indicating that miR-181a is largely expressed from the miR-181ab1 locus in T cells [22, 25]. miR-181a expression is dynamically regulated during the life cycle of a T cell, from their development in the thymus to differentiation and eventually aging in the periphery. Here, we will review the diverse functions of miR-181a in these T cell differentiation pathways and discuss the implication of miR-181a deficiency in T cell responses to infections and vaccinations in older individuals.

## miR-181a and T cell development

**Fig. 1:**
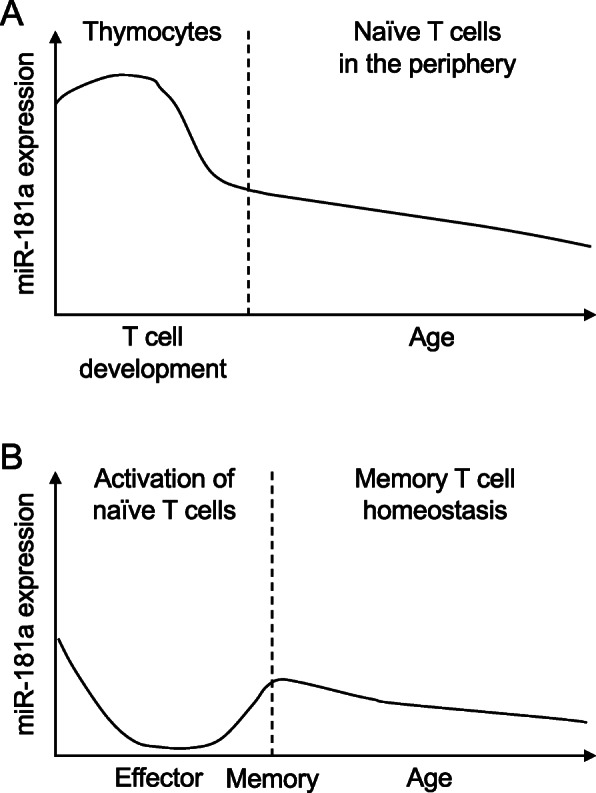
Kinetics of miR-181a expression in T cells. **A** Changes in miR-181a expression during T cell development in the thymus and during aging in the periphery. **B** miR-181a expression changes during T cell activation, differentiation and subsequent T memory cell homeostasis

During T cell development in the thymus, selection of T cells with low to intermediate affinity to self-antigens and elimination of T cells with high affinity are the key for a functional T cell repertoire that is able to maintain central tolerance in the periphery. Therefore, setting the TCR sensitivity to cognate peptide antigens plays an important role in the selection processes. miR-181a was initially described as an intrinsic regulator of TCR signaling thresholds in thymocytes and T cells [26]. miR-181a is highly expressed in CD4 and CD8 double-negative (DN) and double-positive (DP) thymocytes. With differentiation, its expression is downregulated in CD4 or CD8 single-positive (SP) thymocytes and mature T cells in the periphery (Fig. 1), which corresponds to a progressive decrease in TCR sensitivity to cognate antigens [26]. Functionally, miR-181a targets several phosphatases such as SHP2, PTPN22, DUSP5 and DUPS6. PTPN22 inactivates LCK and ZAP70 through dephosphorylation [27]. DUSP5 and DUSP6 are also negative regulators of TCR signaling dephosphorylating extracellular signal–regulated kinase (ERK) in the cytoplasm (DUSP6) as well as the nucleus (DUSP5) [28]. By repressing these multiple negative feedback loops downstream of TCR signaling, miR-181a reduces the activation thresholds and increases TCR sensitivity to cognate antigens [26]. Consequently, ectopic expression of miR-181a in mature T cells augments sensitivity of TCR signaling upon TCR stimulation, as shown by increased phosphorylation of LCK and ERK and increased calcium flux and IL-2 production [22, 26]. Also, miR-181a overexpression in thymocytes promotes the differentiation of DN to DP cells [22, 29]. Conversely, antagonizing miR-181a or its genetic deletion reduces TCR sensitivity and impairs both positive and negative selection of developing DP and SP thymocytes [22, 26, 30]. Accordingly, miR-181a deficiency may fail to delete potentially autoreactive T cells to self-antigens during negative selection in the thymus [31]. Indeed, as compared to wild-type counterparts, mature T cells that developed in miR-181ab1 germline knockout mice showed increased reactivity in response to immunization with self-antigen [30]. However, germline knockout of miR-181ab1 did not cause spontaneous autoimmunity, suggesting that altering T cell repertoire selection is not sufficient to induce disease [30].

Given that each miRNA can target multiple distinct mRNA, miR-181a also controls T cell development by targeting other molecules. miR-181a has been implicated in the development of T cell acute lymphoblastic leukemia (T-ALL) by activating oncogenic NOTCH pathway through repressing its multiple negative regulators [22]. During normal T cell development, NOTCH signaling is known to transcriptionally induce the transcription factor *Tcf7*, also important for T cell development [32]. Therefore, miR-181a regulates T cell development by controlling two major pathways independent of calibrating TCR activation threshold. Disruption of thymic T cell development was also observed in an independent strain of miR-181ab1 knockout mouse [33]. Interestingly, upregulation of phosphatase and defect in TCR-induced ERK phosphorylation were not observed in this miR-181ab1-deficient mouse model. Instead, miR-181a-deficient thymocytes had increased expression of its target PTEN, which inhibits the PI3K-AKT-mTORC1 signaling pathway, an important axis for anabolic metabolism to support cell growth and proliferation [34]. Consequently, the thymic T cell development was impaired in miR-181ab1-deficient mice, with altered cellular metabolism, reduced cell proliferation and increased cell death [33]. The reason for the different biology in the two knockout strains is unresolved and may represent off-target effects.

Controlling TCR signaling by miR-181a is also important for the development of regulatory T cells and several innate-like T cell populations, including invariant natural killer T (iNKT) cells and mucosal associated invariant T (MAIT) cells, that are also generated from double-positive thymocytes [35, 36]. In contrast, miR-181a does not appear to control the generation of γδ T cells which develop from DN thymocytes [37]. miR-181ab1 deficiency impaired de novo generation of thymic regulatory T cells [38], consistent with the requirement of relatively strong TCR signals for their development [39]. Increasing TCR signaling strength through ectopic expression of the Nur77 family member Nr4a2 rescued impaired regulatory T cell development in miR-181ab1-deficient mice, further supporting a mechanistic link [38]. iNKT cells recognize glycolipids through their semi-invariant TCRs, and strong TCR signals are needed for selection during their development [40]. Notably, generation of iNKT cells was severely impaired in miR-181ab1-deficient mice [25, 33]. Administration of agonistic ligand rescued defective iNKT cell generation in miR-181ab1-deficient mice [25], consistent with the notion that miR-181a deficiency increases the activation threshold. In addition to controlling TCR signaling, miR-181a-regulated cellular metabolism also contributes to early iNKT cell development, because genetic deletion of *Pten* restored iNKT cell generation in miR-181ab1-deficient mice [33]. In addition to iNKT cells, miR-181ab1 deficiency impaired generation of MAIT cells that were restored with ectopic expression of their invariant TCRα chain [41], suggesting that TCR signal strength could be also involved during the development of MAIT cells.

## miR-181a and age-related defects in T cell activation

Compared to thymocytes, miR-181a expression is lower in peripheral naïve T cells and further reduced with differentiation and activation with TCR stimulation [26]. Notably, miR-181a levels decline in naïve CD4 T cells from older individuals [42]. Memory CD4 T cells have lower miR-181a expression than naïve CD4 T cells and also tend to have an age-associated decline (Fig. 1) [42]. Similar reduction in miR-181a expression is also found in naïve CD8 T cells from aged humans and mice [15, 43]. In contrast, neonatal naïve CD4 T cells from umbilical cord blood have relatively higher expression of miR-181a than adult naïve CD4 T cells from peripheral blood [44] suggesting that age-associated decline in miR-181a levels may reflect a partially differentiated state of aged naïve T cells, presumably due to homeostatic proliferation.

**Fig. 2:**
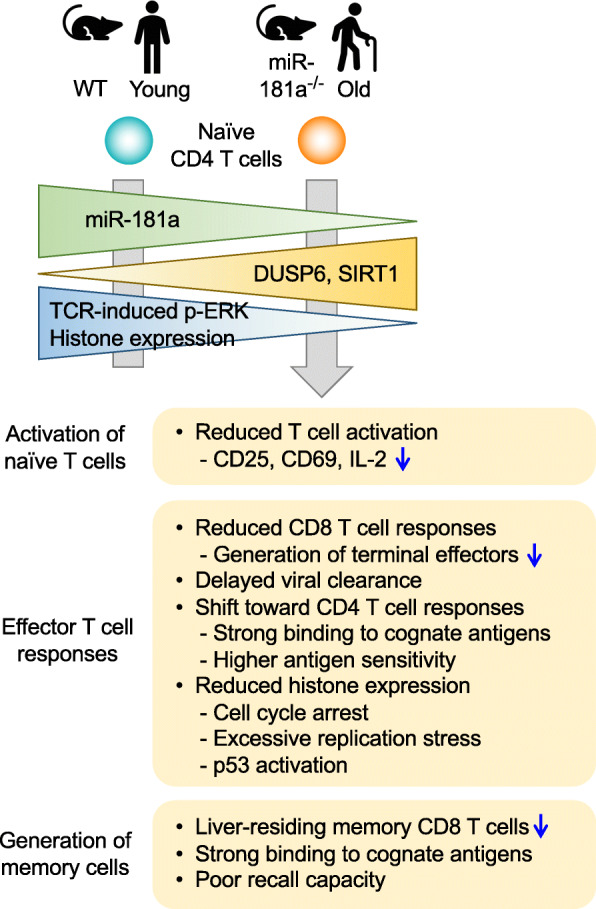
Functional consequences of the age-associated decline in miR-181a expression on viral infection. Summary of shared features of antiviral T cell responses observed in T cell-specific miR-181a-deficient mice and in older individuals during T cell activation, effector differentiation and memory T cell formation

This expression change is functionally important for activation of peripheral naïve T cells (Fig. 2). Consistent with the role of miR-181a in controlling TCR signaling, aged naïve CD4 T cells have a defect in ERK phosphorylation upon TCR stimulation, which is mainly caused by age-associated increase in DUSP6 expression. Conversely, neonatal CD4 T cells have an increased TCR-induced ERK activity due to higher miR-181a expression [44]. With reduced TCR sensitivity, aged naïve CD4 T cells therefore need higher antigenic stimulation to induce activation markers CD69 and CD25 and produce IL-2 at levels comparable with activated young naïve cells [42]. Indeed, increasing the vaccine dose improves vaccine efficacy in older individuals [45–47]. Overexpression of miR-181a, silencing of DUSP6 or pharmacological inhibition of DUSP6 activity restores defective ERK signaling, IL-2 production and proliferative capacity upon TCR stimulation, improving T cell responses of old naïve CD4 T cells [42]. Nearly identical findings have been observed in CD4 T cells from patients infected with hepatitis C virus, with a decline in miR-181a and increase in DUSP6 expression, suggesting that chronic viral infection might induce premature immune aging [48].

## miR-181a and age-related defects in antiviral T cell responses

Given defective T cell activation with reduced miR-181a expression in older individuals, the impact of miR-181a loss in antiviral immune responses has been examined in a mouse model with miR-181ab1 knocked-out in peripheral T cells (Fig. 2) [43]. Conditional deletion of miR-181ab1 after thymic positive selection through the expression of Cre recombinase under the control of distal Lck promoter did not disturb T cell development in the thymus with normal distribution of naïve CD4 and CD8 T cells in the periphery. Consistent with DUSP6 overexpression dampening T cell activation, miR-181a deficiency impaired expansion of antigen-specific CD8 T cells after acute lymphocytic choriomeningitis virus (LCMV) infection in mice [43]. This defect resulted in a delayed viral clearance, recapitulating aged immune responses to viral infection with West Nile virus (WNV) and vaccination with live-attenuated yellow fever virus (YFV) in older individuals [49–54]. Interestingly, LCMV-specific CD4 T cell responses were increased in miR-181a-deficient compared to wild-type mice. This increase was likely a compensatory effect because adoptively transferred, LCMV-specific TCR transgenic wild-type CD4 T cells proliferated more in miR-181a-deficient than in wild-type mice upon LCMV infection. Also, when wild-type and miR-181a-deficient TCR transgenic CD4 T cells together were transferred to the same mouse, the deficient T cells exhibited reduced expansion after LCMV infection, suggesting that increased CD4 T cell immunity shown in miR-181a-deficient mice was due to a delayed viral clearance [43]. Indeed, higher numbers of WNV-specific CD4 T cells were observed in older individuals who had poor viral control [55]. Live-attenuated YFV vaccine also induced prolonged CD4 T cell proliferation in older individuals in parallel to delayed viral clearance [54]. Therefore, the mouse model with miR-181a-deficient peripheral T cells provides insights into the relationship between antiviral CD4 and CD8 T cell immunity and viral control in acute infections with increasing age. Understanding how miR-181a deficiency shapes T cell responses to chronic viral infections or tumors that frequently occur in older individuals will be of interest and remains to be addressed.

Consistent with increasing T cell activation threshold, miR-181a deficiency skewed antiviral CD4 T cell responses toward selecting T cells recognizing antigenic peptides with higher affinity, as shown by their strong binding to cognate peptide/MHC II tetramers [43]. In humans, WNV-specific memory T cells from individuals who were infected at older age bound to the peptide/MHC tetramers more strongly than those from individuals who were infected at younger age [43], providing evidence of repertoire selection. Similarly, mouse miR-181a-deficient CD4 T cells responding to LCMV needed lower amounts of antigenic peptide to elicit half-maximum cytokine production than wild-type cells, further supporting the notion that miR-181a deficiency drives repertoire selection to higher functionality [43]. Interestingly, this repertoire selection was not associated with a contraction in repertoire diversity at the clonal level. Instead, CD4 T cell responses in miR-181a-deficient mice exhibited increased clonal diversity with increased expansion, presumably due to a delayed viral clearance and recruitment of more clones into the response [43]. miR-181a-dependent repertoire selection was also evident during a recall response; miR-181a-deficient memory CD4 T cells displayed stronger binding to tetramers, reduced amounts of antigenic peptides to produce effector cytokines and a more contracted TCR repertoire after secondary infection [43]. In sharp contrast, CD8 T cell response did not show any evidence of repertoire selection towards higher affinity or higher functionality with miR-181a deficiency. Also, the extent of oligoclonality of CD8 T cells responding to LCMV and their repertoire diversity were not different [43], suggesting that expansion of individual CD8 T clones were equally reduced [56]. In summary, the age-related decline in miR-181a expression accounts for delayed viral clearance due to defective CD8 T cell responses. The ensuing altered antiviral CD4 T cell responses are characterized by broadening TCR repertoires with higher avidity and functionality to cognate antigens, trying to overcome defective CD8 T cell responses.

TCR signaling strength has been implicated in directing T cell differentiation, with strong activation signals being required for the development of short-lived terminal effector CD8 T cells and relatively weak TCR signals favoring generation of memory precursor effector cells and central memory T cells [57–59]. Therefore, miR-181a-regulated control of TCR activation threshold can determine T cell fates during a viral infection. Indeed, reduced expansion of CD8 T cells in miR-181a-deficient mice largely resulted from a defect in the differentiation of short-lived effector CD8 T cells, while memory precursors developed normally [43]. In addition, memory CD8 T cells that were generated in miR-181a-deficient mice rapidly acquired central memory phenotypes with higher expression of CD62L and CD27 and were capable of producing multiple cytokines such as IFNγ, TNFα and IL-2 upon restimulation ex vivo. However, they again failed to expand upon subsequent reinfection and did not provide increased protection. Importantly, miR-181a deficiency particularly impaired generation of liver-residing tissue-resident memory CD8 T cells [43]. Tissue-resident memory T cells play an important role in protecting hosts from reinfections at local sites [60]. Also, during CD4 T cell differentiation, miR-181a deletion selectively impaired generation of Th1 cells without altering Tfh cell differentiation after LCMV infection [43]. Similarly, DUSP6 silencing also enhanced IFNγ-producing Th1 cell differentiation under Th1 polarizing condition of aged human CD4 T cells [42]. Similar to the role of signaling strength in effector vs. memory differentiation in CD8 T cells, strong TCR signals are required for generation of more differentiated Th1 CD4 T cells, while weak signals favor Tfh cell differentiation [61, 62]. Thus, reduced miR-181a levels in naïve T cells from older adults impact not only the recruitment of antigen-specific T cell clones into the response but also their differentiation, tissue migration, acquisition of effector functions and recall capacity.

## miR-181a deficiency causes replication stress in T cell responses from old adults

One characteristic feature observed in T cell responses from both miR-181a-deficient mice and older individuals was a reduced expression of core histones after T cell activation [63]. In general, histone loss is one of the hallmarks of aging in several model systems [64, 65], conversely, ectopic expression of histones extends life span in yeast [66]. Histone levels did not differ in unstimulated naïve T cells, suggesting activation-induced defects. Histone transcription is robustly induced at the early S-phase of the cell cycle during proliferation, in order to pack newly synthesized DNA into chromatin [67]. Failure to upregulate histone expression causes DNA replication stress response and stalls cell cycle progression [68, 69]. Consistent with this notion, proliferating T cells accumulated at the early S-phase in miR-181a-deficient mice and older individuals. The p53 and ATR signaling pathways, indicative of replicative stress [70], were abnormally activated, as shown by increased phosphorylation of PRA32 and CHK1, accumulation of DNA damage marker phosphorylated H2aX (γH2aX) and induction of cell cycle inhibitor p21 [63]. This increased replication stress and activation of the p53 pathway may explain recent findings in defective vaccine responses of older individuals [11]. Upon vaccination with the live varicella zoster virus (VZV) vaccine strain, old individuals had expansion of antigen-specific CD4 T cells not different from young adults. However, cell loss after peak responses was accelerated, leaving behind fewer VZV-specific memory CD4 T cells. This contraction correlated with enrichment of gene signatures of cell cycle regulation and DNA repair pathways, indicating a failure in cell cycle regulation [71].

In mice and humans, defects in histone upregulation resulted from overexpression of the miR-181a target SIRT1, a NAD^+^-dependent histone deacetylase [72, 73]. SIRT1 was recruited to histone gene promoters, where it reduced H3K9/14 and H4K16 acetylation locally and consequently suppressed histone gene transcription [74–76]. Importantly, restoring histone expression through inhibiting SIRT1 rescued cell cycle progression, diminished replication stress and improved T cell expansion and viral control [63]. In contrast to these findings in activated T cells, SIRT1 expression is generally thought to decline with age in several tissues including terminally differentiated effector memory CD8 T cells [77, 78], suggesting tissue and cell type specific roles for SIRT1. Increasing SIRT1 expression or activity may improve longevity [79]. Therefore, global SIRT1 inhibition may be harmful, however, transient SIRT1 inhibition immediately after vaccination or in the context of an infection appears to be safe. In this regard, pharmacological SIRT1 inhibitor Ex-527 (Selisistat), which passed phase II clinical trials to treat Huntington’s disease [80], could be used therapeutically to improve vaccine-induced T cell responses in older individuals.

## miR-181a and age-related defects in T cell homeostasis

miR-181a is one of several miRNAs that changes in expression with age in naïve CD8 T cells [15]. Pathway analysis of downstream targets controlled by these miRNAs, including miR-181a, miR-146a, miR-155, let-7f, miR-7 and miR-142, identified a loss of FOXO1 activity with increasing age as significantly enriched. Expression of IL-7R, one of FOXO1 targets, was particularly reduced in aged naïve CD8 T cells [15], which may account for alteration in T cell homeostasis and loss in naïve CD8 T cell numbers with age [81, 82]. In mice, miR-181a alone appears to control homeostatic proliferation of naïve T cells by targeting PTEN [33]. When adoptively co-transferring wild-type and miR-181a-deficient naïve T cells into Rag1-deficient lymphopenic mice, relative proportions and numbers of miR-181a-deficient naïve T cells were severely reduced, presumably due to impaired PI3K signaling [33].

## Age-related defects in transcriptional control of miR-181a

**Fig. 3:**
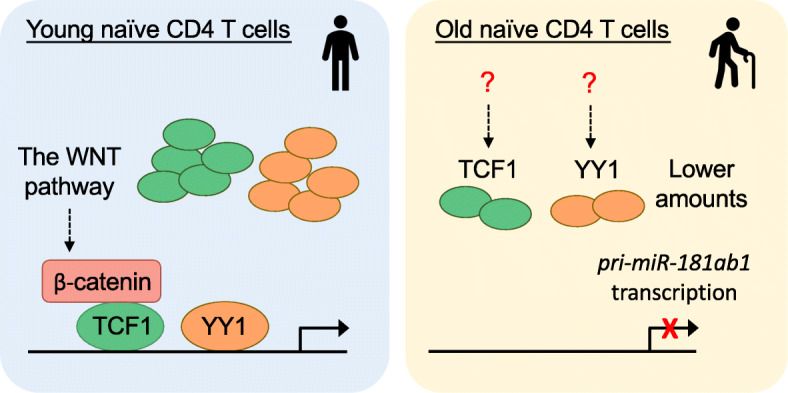
Mechanisms of reduced miR-181a levels in aged T cells. TCF1 and YY1 are the major transcription factors for transcription of pri-miR-181ab1. Age-related reduction of TCF1 and YY1 expression results in a loss of miR-181a expression in old naïve CD4 T cells

In addition to pathways downstream of miR-181a activity, targeting the upstream mechanism underlying the age-related miR-181a loss has the potential of improving defective T cell responses in older individuals. Like all miRNA genes, miR-181a is transcribed as a primary miRNA (pri-miRNA), encompassing miR181a and b, and subsequently processed into precursor miRNAs (pre-miRNAs) in the nucleus and mature miRNA in the cytoplasm. The age-associated decline in miR-181a levels results from reduced transcription of pri-miR-181ab1 [73]. Analysis of putative enhancer regions of the miR-181ab1 locus identified YY1 and TCF1 as major transcription factors inducing pri-miR-181ab1 transcription. Reduced expression of YY1 and TCF1 caused the age-related decline in pri-miR-181a transcription (Fig. 3) [73, 83]. Accordingly, restoring YY1 or TCF1 expression upregulated miR-181a and thereby improved T cell responses from older adults.

YY1 has been implicated in many biological processes such as cell growth and development, where it functions as a transcriptional activator or repressor depending on the interaction with other cofactors [84, 85]. Along with the age-related decline in YY1 expression, naïve CD8 T cells from the elderly show reduced chromatin accessibility to YY1 binding sites at gene promoters [86, 87]. YY1 silencing had only minimal effects on the transcriptome of unstimulated naïve CD4 T cells beyond miR-181a expression [73]. Since YY1 is involved in controlling differentiation and function of Th1, Th2 and regulatory T cells in mice [88, 89], the reduced expression may account for age-related differences in differentiation and effector functions of activated T cells.

The age-related decline in TCF1 expression is particularly intriguing. TCF1 is a transcription factor and an effector molecule downstream of the WNT/β-catenin pathway [90]. Inhibition of GSK3β stabilizes β-catenin and the subsequent β-catenin/TCF1 complex induces TCF1-dependent gene expression including *TCF7* itself. Pharmacological inhibition of GSK3β induced both TCF1 and pri-miR-181ab1 transcription and consequently improved T cell activation in older adults [83]. While important for T cell development [32], TCF1 also maintains a less differentiated stem-like cell state of peripheral T cell responding to acute and chronic viral infections and cancer [91–93]. Interestingly, the reduced expression of TCF1 in older adults persists throughout a T cell response, contributing to the development of terminally differentiated proinflammatory effector cells rather than memory precursor cells [17, 94]. TCF1 declines after activation in effector cells, but is re-expressed in memory cells, albeit at a lower level compared to naïve cells. Whether TCF1 drives pri-miR-181ab1 expression in memory T cells and its functional consequences remains to be addressed.

## Conclusions

miR-181a expression is dynamically regulated during T cell development in the thymus as well as during T cell differentiation in the periphery. It is abundantly expressed in the thymus, where miR-181a facilitates thymic development of conventional T cells, regulatory T cells, iNKT cells and MAIT cells by lowering TCR activation thresholds through repressing multiple phosphatases. Albeit at lower levels in the periphery, miR-181a similarly increases TCR sensitivity to antigens and promotes activation of peripheral T cells. Due to a loss of miR-181a expression, naïve T cells from older individuals fail to respond properly to T cell stimulation, exemplified by suboptimal responses particularly for T cells with lower affinity to antigens or for stimulation with weaker antigenic signal.

Emerging data also indicate that miR-181a is involved in other pathways important for T cell responses. Controlling cellular metabolism by repressing PTEN is crucial for the development of T cells and iNKT cells [33]. Given that PTEN is targeted by multiple miRNAs including miR-21 whose expression is increased with age [17], contribution of miR-181a to PTEN expression in aged T cells appears to be limited. The NOTCH pathway is activated by miR-181a expression in T cells, which contributes to T-ALL development [22]. Whether the NOTCH pathway is attenuated in T cells from old adults is undetermined. Interestingly, a mouse model with miR-181ab1 deficiency in T cells found that miR-181a is controlling many aspects of antiviral T cell responses, including T cell expansion, repertoire selection, effector and memory T cell development and recall responses, thereby recapitulating many age-related differences in human T cell responses [43]. Some of the phenotypes are clearly related to the role of miR-181a in initial T cell activation signals and the ensuing effects on T cell differentiation pathways. Others cannot be explained by TCR activation threshold, such as defects in T cell proliferation and failed generation of tissue-resident memory T cells with miR-181a deficiency. In this regard, SIRT1 is another important target of miR-181a in the context of aging. SIRT1 expression is increased in naïve T cells of miR-181a-deficient mice as well as older individuals. By repressing histone upregulation during proliferation, it leads to cell cycle arrest and excessive replication stress, thereby inhibiting T cell proliferation [63].

These insights provide potential targets for therapeutic interventions to restore impaired antiviral and vaccine responses in older individuals [95]. Targeting miR-181a or miR-181a-regulated pathways could improve T cell activation and function. Enhancing T cell activation by silencing DUSP6 was not sufficient to rescue proliferative defects of miR-181a-deficient T cells after LCMV infection [43], suggesting cooperative effects of multiple phosphatases. Inhibition of SIRT1 activity in old T cells is promising, as it improves T cell proliferation by restoring histone upregulation and diminishing excessive replication stress. The finding that an age-related decline of YY1 and TCF1 and the consequently reduced pri-miR-181ab1 transcription accounts for the low miR-181a levels is intriguing [73, 83]. Indeed, ectopic overexpression of YY1 or TCF1, or increasing WNT signaling improves T cell activation through induction of pri-miR-181ab1.

## Data Availability

Not applicable.

## Abbreviations

3′ UTR: 3′ untranslated region
DN thymocyte: CD4 and CD8 double negative thymocyte
DP thymocyte: CD4 and CD8 double positive thymocyte
ERK: Extracellular signal–regulated kinase
iNKT cell: Invariant natural killer T cell
LCMV: Lymphocytic choriomeningitis virus
MAIT cell: Mucosal associated invariant T cell
MHC: Major histocompatibility complex
miRNA: MicroRNA
NF-κB: Nuclear factor-κB
pre-miRNA: Precursor miRNA
pri-miRNA: Primary miRNA
SP thymocyte: CD4 or CD8 single positive thymocyte
T-ALL: T cell acute lymphoblastic leukemia
TCR: T cell receptor
WNV: West Nile virus
YFV: Yellow fever virus
